# A rapid, efficient, and facile solution for dental hypersensitivity: The tannin–iron complex

**DOI:** 10.1038/srep10884

**Published:** 2015-06-03

**Authors:** Dongyeop X. Oh, Ekavianty Prajatelistia, Sung-Won Ju, Hyo Jeong Kim, Soo-Jin Baek, Hyung Joon Cha, Sang Ho Jun, Jin-Soo Ahn, Dong Soo Hwang

**Affiliations:** 1 POSTECH Ocean Science and Technology, Pohang University of Science and Technology (POSTECH), Pohang 790-784, Korea; 2 School of Interdisciplinary Bioscience and Bioengineering, Pohang University of Science and Technology (POSTECH), Pohang 790-784, Korea; 3 Dental Research Institute and Department of Biomaterials Science, School of Dentistry, Seoul National University, Seoul 110-749, Korea; 4 Department of Chemical Engineering, Pohang University of Science and Technology (POSTECH), Pohang 790-784, Korea; 5 Department of Dentistry, Anam Hospital, Korea University Medical Centre, Seoul 136-705, Korea; 6 School of Environmental Science and Engineering, Division of Integrative Biosciences and Biotechnology, Pohang University of Science and Technology (POSTECH), Pohang 790-784, Korea

## Abstract

Dental hypersensitivity due to exposure of dentinal tubules under the enamel layer to saliva is a very popular and highly elusive technology priority in dentistry. Blocking water flow within exposed dentinal tubules is a key principle for curing dental hypersensitivity. Some salts used in “at home” solutions remineralize the tubules inside by concentrating saliva ingredients. An “in-office” option of applying dense resin sealants on the tubule entrance has only localized effects on well-defined sore spots. We report a self-assembled film that was formed by facile, rapid (4 min), and efficient (approximately 0.5 g/L concentration) dip-coating of teeth in an aqueous solution containing a tannic acid–iron(III) complex. It quickly and effectively occluded the dentinal tubules of human teeth. It withstood intense tooth brushing and induced hydroxyapatite remineralisation within the dentinal tubules. This strategy holds great promise for future applications as an effective and user-friendly desensitizer for managing dental hypersensitivity.

Approximately 15% to 57% of adults experience dental hypersensitivity, which is a frequent and sharp dental pain, for which a specific and effective nonoperative treatment is yet to be developed^1^,^2^,^3^. Human teeth are primarily composed of highly mineralised hydroxyapatite (HA)-containing enamel and less mineralised HA-containing dentin with 2–5-μm dentinal tubules (Fig. 1a)^2^,^3^. The pain stems from fluid flow within the exposed dentinal tubules that occurs in response to thermal, evaporative, tactile, osmotic, chemical, and electrical stimuli^2^,^3^.

The key to minimise or cure dental hypersensitivity is by occluding the exposed dentinal tubules^4^,^5^. In this paper, we discuss “in-office” and “at-home” nonoperative options^5^,^6^. The “in-office” option involves application and curing of a dentin sealant, a dense polymer resin primarily composed of glutaraldehyde and (hydroxyethyl)methacrylate (40 wt% of aqueous solution) (e.g. Gluma (Heraeus Kulzer, Hanau, Germany), directly on the exposed tubules of dentin^6^. This technique immediately shows good effects. However, such effects are localized to defined sore spots and are diminished if the polymer peels off^5^,^7^. In addition, Gluma needs to be used strictly on a doctor’s advice because glutaraldehyde is toxic and its efficacy depends on a clinician’s skill^5^,^8^. Some salts such as nitrate, fluoride, or oxalate in toothpaste and mouthwash indirectly occlude the dentinal tubules by regenerating HA [Ca_5_(PO_4_)_3_(OH)] mineral from salivary ingredients such as calcium(II) (Ca^2+^) and phosphate (PO_4_^–^) ions^9^,^10^,^11^,^12^,^13^,^14^. However, such effects are not immediate and cannot endure mechanical stress^3^,^5^. Recent studies showed that a mussel-inspired polydopamine coating markedly remineralizes HA in the tubules by binding with Ca^2+^ ions^15^,^16^. However, the polydopamine coating takes at least 6 h to set on the substrate, and its precursor (dopamine) is relatively expensive and has low chemical stability because of its propendency to oxidation^17^,^18^,^19^,^20^.

Tannins, which are common in wines and teas, have been occasionally used as tooth-coating materials^21^,^22^,^23^,^24^ (Fig. 1b). Among the tannins, an emerging tannic acid–iron(III) [TA/Fe^3+^] coating method suggests insights for developing a novel desensitizer^25^,^26^,^27^. In the presence of Fe^3+^ in a mild aqueous solution, many pyrogallol groups in TA form complexes with the Fe^3^ ions and adhere to various substrates; this reaction forms a robust and stable TA/Fe^3+^ complex film on the substrate within only 30 s^25^. In addition, pyrogallol groups of TA can strongly capture calcium ions, which are a key component of HA^26^,^28^. We observed that our modified TA/Fe^3^ coating sealed the microholes of a filter membrane with a geometry similar to that of dentinal tubules (Supplementary Fig. S1).

In this paper, we report a facile, rapid, and efficient dentinal tubule-occluding method that can overcome the problems of dentin sealants and HA-inducing agents. After only 4 min of dipping in a very dilute TA/Fe^3+^ aqueous solution (0.5 g/L), the tubule entrances in the tooth slices were narrowed or covered by the TA/Fe^3+^-complex film. This film was mechanically stable to 1,000 strokes of tooth brushing. This coating also induced relatively quick remineralisation of HA by pyrogallol-mediated Ca absorption in the tubules during incubation in classic artificial saliva.

## Results and Discussion

**Figure 1:**
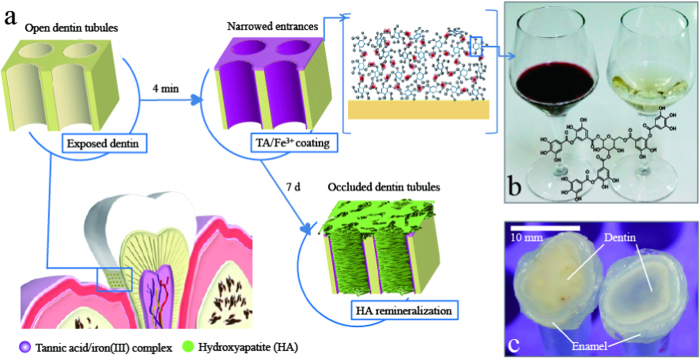
The tannic acid–iron(III) complex-mediated dentin desensitizer. (**a**) A schematic of the tannic acid–iron(III) [TA/Fe^3+^] complex-mediated desensitizing process for occluding the exposed dentinal tubules. (**b**) Wine has a high tannin content. (**c**) The dentin colour changes to a light purple after undergoing the TA/Fe^3+^ coating process.

After obtaining the consent of the patients, human molars were extracted by the standard procedures. The molars were cut perpendicularly to the longitudinal axis into 1-mm slices. The round tooth slices contained yellow dentin in the middle and ivory enamel around the outer edge (Fig. 1c)^10^,^29^. Five types of tooth slice samples were prepared: (1) uncoated, (2) diluted Gluma–coated (0.5 g/L), (^3^) original Gluma-coated, (4) TA only-coated, and (5) TA/Fe^3+^-coated. The uncoated tooth slices were immersed in distilled (DI) water for 4 min. For the TA/Fe^3+^ coating, the tooth slices were dipped in a mixture of TA (0.4 g/L) and FeCl_3_ (0.1 g/L) (pH 8) for 1 min. The dipping process for each slice was repeated 4 times. The TA/FeCl_3_ solution was newly prepared each time because we observed that the coating solution rapidly produced a TA/Fe^3^ complex film within the first 1 min. For the TA only coating, the tooth slices were immersed in the TA solution (0.5 g/L; pH 8) for 4 min. For the Gluma coating, diluted Gluma or original Gluma was applied to the tooth slices and cured in the atmosphere in accordance with the manufacturer’s instructions^6^,^7^. The concentration of the diluted Gluma was adjusted to 0.5 g/L, which is comparable to the TA/Fe^3+^ coating solution. The surface of the whole coated tooth slices was abraded by 1,000 brush strokes at 200 g force and 60 strokes/min using a conventional tooth brushing simulator.

After applying the TA/Fe^3^ coating, the dentin colour changed to purple, whereas the enamel remained white (Fig. 1). Purple is the typical colour of the iron-pyrogallol complex; this finding suggests that TA/Fe^3+^ film is predominantly deposited on the dentin surface^25^,^26^. Compared to enamel, dentin generally has a higher concentration of proteins, which provide cross-linkable functionality to TA^23^,^24^. This is an encouraging fact for dental application because the colour of enamel, which is the visible part of the tooth, is an important cosmetic issue. The exposed dentin that could be dyed after the TA/Fe^3+^ coating is primarily located on cervical area of the tooth, which is a narrow region adjacent to the pink gum tissue. Thus, it is expected that the colour change would not be conspicuous. However, before this coating can be used on patients, research is required on the acceptability of the colour difference between the TA/Fe^3^-coated area and uncoated teeth (or gum)^30^. The TA-only coating, the diluted Gluma coating, and the original Gluma coating did not show a perceptible colour change^7^,^22^,^29^.

**Figure 2:**
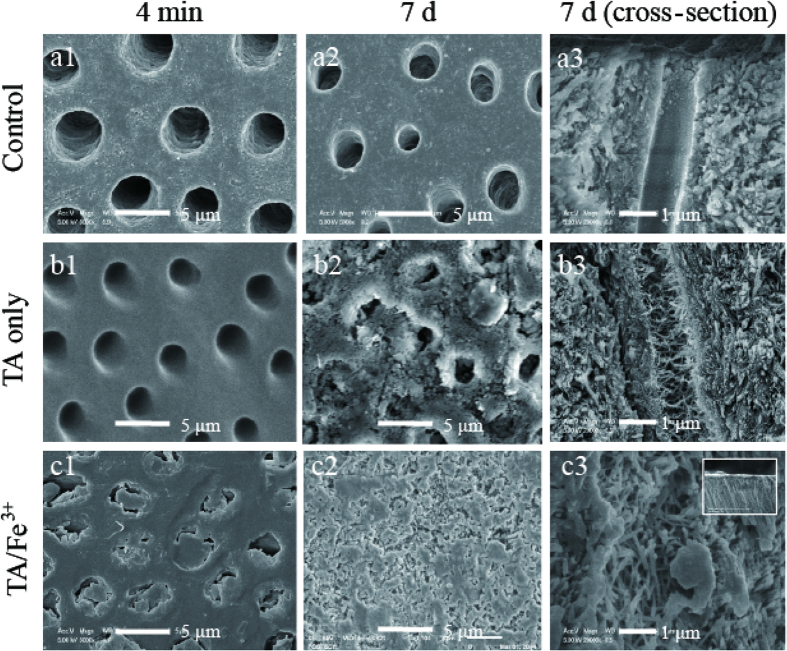
The occlusion effects of the TA/Fe^3+^ complex on the dentinal tubules of the human tooth. **** Scanning electron microscopy images of (**a1–a3**) the uncoated tooth slices, (**b1–b3**) the TA/Fe^3+^-coated tooth slices, and (**c1–c3**) the TA only-coated tooth slices (dentin area). (**a1,b1,c1**) The top view images of a tooth slice before its immersion in the artificial saliva. (**a2,b2,c2**) The top view images after 7 days of immersion in the artificial saliva. (**a3,b3,c3**) The cross-sectional images of the tooth slice after 7 days of immersion in the artificial saliva. The inset in (**c3**) shows a lower magnification of the sample; most of the area is occluded with anisotropic hydroxyapatite minerals.

Gluma is a well-established dentin sealant that directly occludes the dentinal tubules by glutaraldehyde-mediated coupling with the proteins of dentin^5^,^29^. The TA/Fe^3^ coating was likewise expected to function as a sealant because it was deposited in high concentrations in the microholes of the filter membrane (Supplementary Fig. S1). To evaluate the occlusion effect of the TA/Fe^3+^ coating, the topology of the tooth slice samples was observed by scanning electron microscopy (SEM) (Fig. 2). The uncoated sample showed numerous defined and regular tubules (i.e. 2- to 4-μm diameter) that penetrated the dentin perpendicular to the surface. The TA-only coating did not show significant topological occlusion of the dentinal tubules, as do general HA-inducing agents such as nitrate and polydopamine^9^,^10^,^11^,^12^,^13^. However, the TA/Fe^3+^ coating narrowed the tubule entrances, but did not fill the middle of the tubules. This indicates that the TA/Fe^3+^ complex film was deposited to a thickness of several micrometers within only 4 min. The deposition rate of the TA/Fe^3+^ film was remarkably rapid, compared to various self-assembly coatings (1–100 nm/h) such as the polydopamine method^15^,^16^,^17^ and the layer-by-layer method^31^. The original Gluma coating totally covered the tubule entrances, but the diluted Gluma coating did not show any topological occlusion (Supplementary Fig. S3). Thus, because of the lower concentration of the TA/Fe^3+^ coating (compared to Gluma^5^,^6^,^7^; approximately 40 wt%), the TA molecules in the TA/Fe^3+^ solution were more efficiently piled on the dentin, compared to glutaraldehyde and/or (hydroxyethyl)methacrylate in Gluma^8^,^29^. This suggests that, compared to the glutaraldehyde-mediated coupling of Gluma, the pyrogallol–iron complex more efficiently achieved cross-linking of the ingredient molecules in an aqueous environment because of the high binding energy of the pyrogallol-iron complex in water^25^,^31^,^32^,^33^,^34^. Furthermore, the fact that the TA/Fe^3+^ complex film remained after mechanical brushing is encouraging because the oral environment is under various mechanical stresses such as chewing, brushing, and temperature-driven air pressure. This good mechanical stability is probably because pyrogallol groups have a strong adhesion to the substrate and a high density of cross-links with iron^22^,^25^,^26^,. Tunicates are sessile marine organisms that glue their torn tissues under the sea. A clue to this adhesion lies in the pyrogallol group^34^.

Polydopamine, nitrate, and oxalate remineralize HA by binding with calcium ions in saliva^9^,^10^,^11^,^12^,^13^,^35^. The pyrogallol group of TA binds calcium ions in aqueous environments^27^,^28^; this was confirmed by x-ray photoelectron spectroscopy (XPS) (Supplementary Fig. S4)^16^. Thus, the TA/Fe^3+^ complex in the tubule was expected to occlude the dentinal tubules by remineralizing HA mineral in saliva. After immersion in artificial saliva for 7 days, the topology of the uncoated tooth slices, the TA only-coated tooth slices, and the TA/Fe^3^-coated tooth slices was observed using SEM (Fig. 2). The dentinal tubules and the surface of the TA/Fe^3+^-coated tooth slice were densely and uniformly filled with needle-like minerals. However, the TA only-coated tooth slices showed sparse needle-like minerals, and the uncoated tooth slices did not show needle-like minerals.

**Figure 3:**
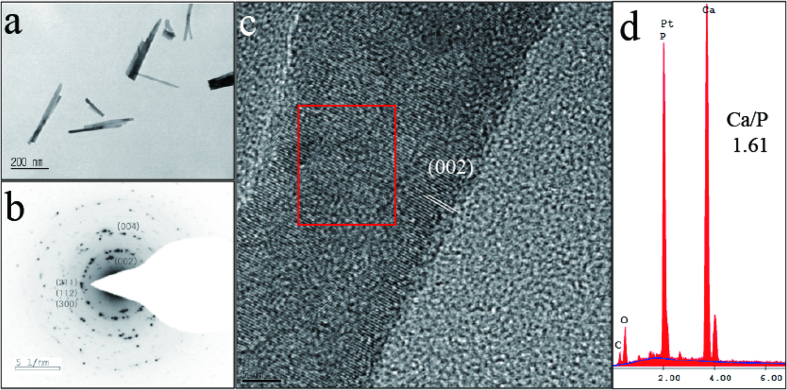
Pyrogallol-mediated hydroxyapatite remineralisation. (**a**) Transmission electron microscopy micrograph of fragmented anisotropic minerals from a hydroxyapatite (HA)-mineralised tannic acid–iron(III) (TA/Fe)-coated tooth slice. (**b**,**c**) The selected area electron diffraction (SAED) pattern and the inset red box indicates the area where SAED was taken. (**d**) Energy dispersive x-ray spectrum of the HA-mineralized TA/Fe^3+^-coated tooth slice. Ca/P = calcium/phosphorus.

The needle-like mineral was identified as HA, based on energy dispersive x-ray (EDX) and transmittance electron microscopy (TEM)^16^ (Fig. 3). The EDX analysis revealed that the calcium/phosphorus (Ca/P) ratio of the needle-like minerals was 1.61, which is comparable to the 1.66 theoretic value of HA^12^,^13^,^14^,^15^,^16^.

The needle-like minerals of the TA/Fe^3+^-coated sample were fragmented by ultrasonication in ethanol, and the fragments were characterized by TEM. The need-like mineral generally had a length of 100–300 nm and a 10–20 aspect ratio. Its selected area electron diffraction pattern gave the characteristic HA peaks: (002), (004), (112), (211), and (300). The high-resolution TEM image showed that the (002) crystal plane is perpendicular to the c-axis (i.e. longitudinal axis) of HA^16^. This suggests that the anisotropic growth of needle-like HA occurred along (002). Polydopamine reportedly induces the anisotropic growth of HA toward the c-axis^16^. Pyrogallol groups of TA are similarly likely to induce the anisotropic growth of HA. The absence of HA minerals in the uncoated sample suggests that the pyrogallol groups involve HA nucleation. The higher density of HA on the TA/Fe^3+^ coating, compared to the density of HA on the TA only coating, is probably because TA molecules of the TA/Fe^3+^ mixture solution are more highly deposited on the substrate than in the TA-alone solution.

To exclude other possible factors that affect HA remineralisation (e.g. the confined space of the tubule, Ca-binding groups of dentin proteins, and long incubation time), TA/Fe^3^-coated polystyrene (PS, a hydrophobic and inert polymer)^25^ and uncoated PS films were incubated in the artificial saliva for 1 day and their surfaces were observed using SEM (Supplementary Fig. S5). The TA/Fe^3^-coated PS film was prepared in the same manner as the TA/Fe^3+^-coated tooth slices. The TA/Fe^3^-coated PS surface was filled with flower-shaped HA minerals, whereas the uncoated PS surface did not show such minerals. The fragmented HA mineral was observed by TEM; they were also needle-like anisotropic HA. The results reconfirmed that pyrogallol groups of TA mediated the anisotropic formation of the HA mineral. The pyrogallol groups of TA overall contributed to the adhesion to the substrate and highly anisotropic HA formation by absorbing Ca^2+^ ions. Thus, the highly anisotropic HA growth on the PS surface suggests that the TA/Fe^3+^ coating provides insights in creating organic-inorganic hybrid biomaterials.

**Figure 4:**
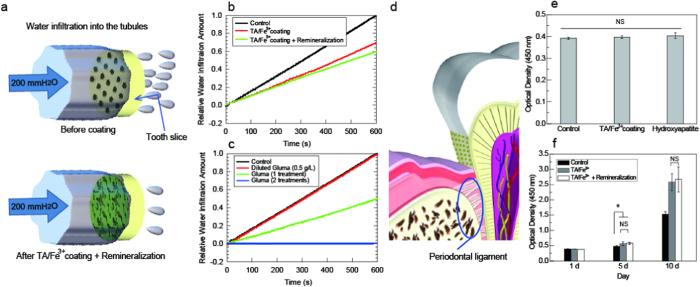
Dentinal fluid flow test and human periodontal ligament cell culture. (**a**) Evaluation of dentinal tubule occlusion. The water infiltration amount in (**b**) the tannic acid/iron(III) [TA/Fe^3+^]-coated tooth slices and (**c**) the Gluma-treated tooth slices at 200 mmH_2_O for 600 s. (**d**) Schematic of the human periodontal ligament tissue. Evaluation of the human periodontal ligament (hPDL) cell (**e**) attachment and (**f**) proliferation on TA/Fe^3+^-coated cell culture dish and hydroxyapatite remineralised cell culture dish (n = 3; the mean ± the standard error of the mean).NS = not significant (*P* > 0.05).* *P* < 0.05.

Dental hypersensitivity originates from neural stimulation by fluid flow within the dentinal tubules that is driven by physiological pulpal pressure of approximately 200 mmH_2_O^36^,^37^ (Fig. 4a). To study the *in vitro* ability of the TA/Fe^3^ complex and pyrogallol-induced HA to restrain dentinal fluid flow, the amount of water infiltration in a neat tooth slice for 10 min at 200 mmH_2_O was first measured as the standard^36^,^37^(Fig. 4b). The water infiltration amounts after the TA/Fe^3+^ coating and after incubation in artificial saliva for 7 days were again measured. The TA/Fe^3+^ coating reduced the water infiltration to approximately 68% of the standard tooth slice, which suggests that the topologically observed TA/Fe^3+^ complex actually restrained the dentinal fluid flow. Incubation in the artificial saliva reduced the water infiltration to approximately 59% of the standard tooth slice. The results suggest that the regenerated HA provided additional dentinal fluid blockage.

The water infiltration amounts were measured after applying the original Gluma and diluted Gluma treatments (Fig. 4c). The diluted Gluma did not show dentinal fluid blockage, as revealed by the SEM study. A single treatment of the original Gluma reduced the water infiltration to approximately 53% of the standard, which was of a grade equivalent or somewhat lower than water infiltration of the remineralized TA/Fe^3+^-coated tooth slice. This thus demonstrated that the TA/Fe^3^ coating provided favourable tubule occlusion. Double treatments of the original Gluma totally blocked water infiltration. However, Gluma is generally effective only on a specific and localized area and its performance depends on a clinician’s skill^5^,^6^,^7^,^38^. Many patients cannot exactly define their sore teeth because the pain spreads throughout several teeth^1^,^5^. It is also dangerous for patients to use glutaraldehyde-containing chemicals as a treatment without a specialist’s assistance^4^,^5^,^7^,^8^,^38^. However, tannin has always been present in the diet of humans and animals, and its safety has been proven^21^,^22^. Low cytotoxicity of the TA/Fe^3+^ complex to yeast cells has recently been reported^39^.

A healthy periodontal ligament tissue, a tissue adjacent to dentin, tightly anchors the tooth to the alveolar bone^41^ (Fig. 4d). Dentin can be otherwise exposed to the outside because of a reduced gum level. Thus, to show quantitatively the physiological adaptation and affinity of the human periodontal ligament (hPDL) cell (i.e. fibroblast) on the TA/Fe^3^ coating, the attachment and proliferation of the hPDL cell in 3 types of culture dishes were investigated (Figs. 4e,f): uncoated, TA/Fe^3+^-coated, and HA-remineralized culture dishes. In the cell attachment test, there were no significant differences between both types of coated surfaces and the control. The proliferation rate of the hPDL cells on the TA/Fe^3^ coating was comparable to that of the remineralized HA surface but notably higher than that of the control (Fig. 4f). Good proliferation of the hPDL cells on the HA surface has been well reported^40^. This result demonstrates that the TA/Fe^3+^ coating also provides low-toxic, physiologically adaptive, and physiologically affinitive environment for the hPDL cell. This is encouraging because hPDL cells have important functions in the repair and regeneration of periodontal tissues^41^. Thus, the TA/Fe^3+^ coating may improve the regenerative competency of the periodontal ligament tissue and reduce further exposure of dentin to the outside.

We found the following advantages of the TA/Fe3+ coating as a novel dentin desensitizing agent, compared to existing desensitizers. First, the TA/Fe^3+^ coating was rapid, efficient, and facile in occluding the tubules. Even diluted concentration of TA and iron ions spontaneously formed a mechanically stable complex film on the microsized tubules within only 4 min. Second, the TA/Fe^3+^ coating was facile and independent of a patient’s skill. Thus, it can be commercialized as an “at-home” desensitizer product, as are tooth paste and mouthwash. Third, this process does not coat a localized area, as do some conventional treatments, but it coats the whole area of a tooth exposed to the TA/Fe^3+^ complex. Thus, this TA/Fe^3+^ complex-mediated strategy would be effective when a patient has difficulty identifying sore areas. Fourth, TA is a sustainable, eco-friendly, economical, and biocompatible material because it can be extracted from timber or fruit residues and administered in many foods such as wines, teas, and fruits. In addition, this coating showed low cytotoxicity and good adhesion to the hPDL cell, which can reduce further dentin exposure to the outside.

In summary, we reported an immediate and effective dentin desensitizer, a TA/Fe^3+^ complex-based coating, as a dentinal tubule-occluding material. As a dip coating, the spontaneously formed TA/Fe^3+^ complex coating narrowed or covered the dentinal tubules entrance. The complex also endured mechanical brushing, which is a possible stressor in the oral environment. Furthermore, with incubation in artificial saliva, the complex induced remineralisation inside the exposed tubules by reconstructing Ca^2+^ and PO_4_^–^ ions from the saliva. Pyrogallol moieties in TA were responsible for the nucleation of the anisotropic growth of HA. In addition, the TA/Fe^3+^ coating showed low-toxicity and good physiological affinity to the periodontal ligament, which is a tissue adjacent to dentin. The TA/Fe^3+^ complex-mediated coating overall is a solution for treating dentin hypersensitivity and for developing anisotropic HA-embedded hybrids that are applicable to emerging biomedical materials.

## Methods

Tannic acid, iron(III) chloride, sodium hypochlorite, and calcium chloride were purchased from Sigma-Aldrich. Phosphates, and tris(hydroxymethyl)aminomethane (Tris)–hydrochloric acid (HCl) were purchased from EM Science (Gibbstown, NJ, USA). Phosphoric acid etching gel was purchased from the 3 M Company (St. Paul, MN, USA). Gluma was purchased from Heraeus Kulzer (Hanau, Germany).

Human molars were collected in accordance with the guidelines approved by the institutional review board (IRB) of the Korea University Medical Centre (Seoul, Korea). The teeth were treated with 3% sodium hypochlorite to remove bacteria, and rinsed with phosphate buffered saline (PBS). The tooth crown containing enamel and dentin were cut perpendicular to the longitudinal axis into 1-mm thick tooth slices with a diameter of approximately 8−10 mm using a low speed diamond saw. The tooth slices were acid-etched with phosphoric acid etching gel for 30 s, and then rinsed with sufficient DI water.

Artificial saliva was prepared, as follows: pH 7.6 buffer 50 mM Tris–HCl with 2.58 mM calcium chloride, 1.55 mM phosphates, and 180 mM sodium chloride. Tooth slice samples were immersed in the artificial saliva solution, air-tight sealed, and incubated at 37 °C statically for 7 days. After the immersion, the tooth slides were removed from the solution, and then rinsed with running DI-ionized water for 50 s.

## Additional Information

**How to cite this article**: Oh, D. X. *et al.* A rapid, efficient, and facile solution for dental hypersensitivity: The tannin-iron complex. *Sci. Rep.* **5**, 10884; doi: 10.1038/srep10884 (2015).

## Supplementary Material

Supplementary Information
