# Supplementary material for: A rapid, efficient, and facile solution for dental hypersensitivity: The tannin–iron complex
Source: Sci Rep. 2015 Jun 3;5:10884. doi: 10.1038/srep10884 (PMC4454135; doi:10.1038/srep10884)
Supplement: Supplementary Information [file srep10884-s1.pdf]

Supplementary Information

A rapid, efficient, and facile solution for dental hypersensitivity: The tannin–iron complex

Dongyeop X. Oh[1], Ekavianty Prajatelistia[2], Sung-Won Ju[3], Hyo Jeong Kim[4], Hyung Joon Cha[4], Soo-Jin Baek[3], Sang-Ho Jun[5], Jin-Soo Ahn[3], & Dong Soo Hwang[1,2,6]

[1] POSTECH Ocean Science and Technology, Pohang University of Science and Technology (POSTECH), Pohang 790-784, Korea. [2] School of Interdisciplinary Bioscience and Bioengineering, Pohang University of Science and Technology (POSTECH), Pohang 790-784, Korea. [3] Dental Research Institute and Department of Biomaterials Science, School of Dentistry, Seoul National University, Seoul 110-749, Korea. [4] Department of Chemical Engineering, Pohang University of Science and Technology (POSTECH), Pohang 790-784, Korea. [5] Department of Dentistry, Anam Hospital, Korea University Medical Centre, Seoul 136-705, Korea. [6] School of Environmental Science and Engineering, Division of Integrative Biosciences and Biotechnology, Pohang University of Science and Technology (POSTECH), Pohang 790-784, Korea. Correspondence and requests for materials should be addressed to S.H.J (email: omfs.junsang@gmail.com), J.S.A (email: ahnjin@snu.ac.kr), or to D.S.H (email: dshwang@postech.ac.kr).

Materials

Polytetrafluoroethylene (PTFE) membrane filter (0.2 μm pore size, Whatman PLC, UK) were all cleaned in methanol just before use. Polystyrene (PS) film was prepared by cutting a PS Petri dish.

TA/Fe^{3+} coating on PS film, PS 24 wells cell culture plate, and PTFE membrane

PS film, PS 24 wells cell culture plate, and PTFE membrane were coated with the TA/Fe^{3+} complex film as follows. TA (0.4 g/L) and iron(III) chloride (0.1 g/L) were dissolved and the pH of this solution was subsequently raised by adding 10X Tris buffered saline (TBS) to pH 8 solutions. Tooth slices were immersed in the TA/Fe^{3+} solution for 1 min, and this dipping process for each slice was repeated 4 times. The TA/$FeCl_3$ solution was newly prepared each time because we observed that the coating solution rapidly produced a TA/Fe^{3+} complex film within the first 1 minute.

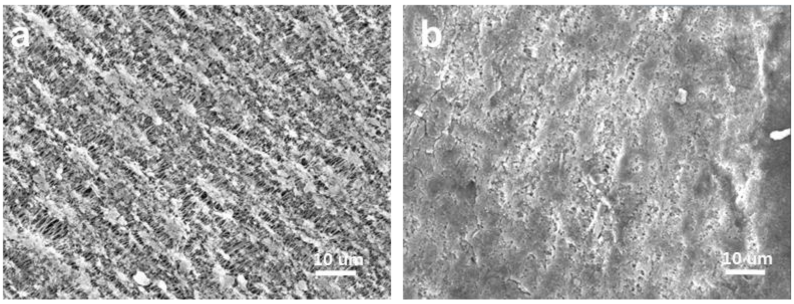

Figure S1. SEM images of a polytetrafluoroethylene (PTFE) filter membrane microholes (**a**) without TA/Fe^{3+} coating and (**b**) with TA/Fe^{3+} coating.

The TA/Fe^{3+} coating sealed the microholes on the PTFE filter membrane.

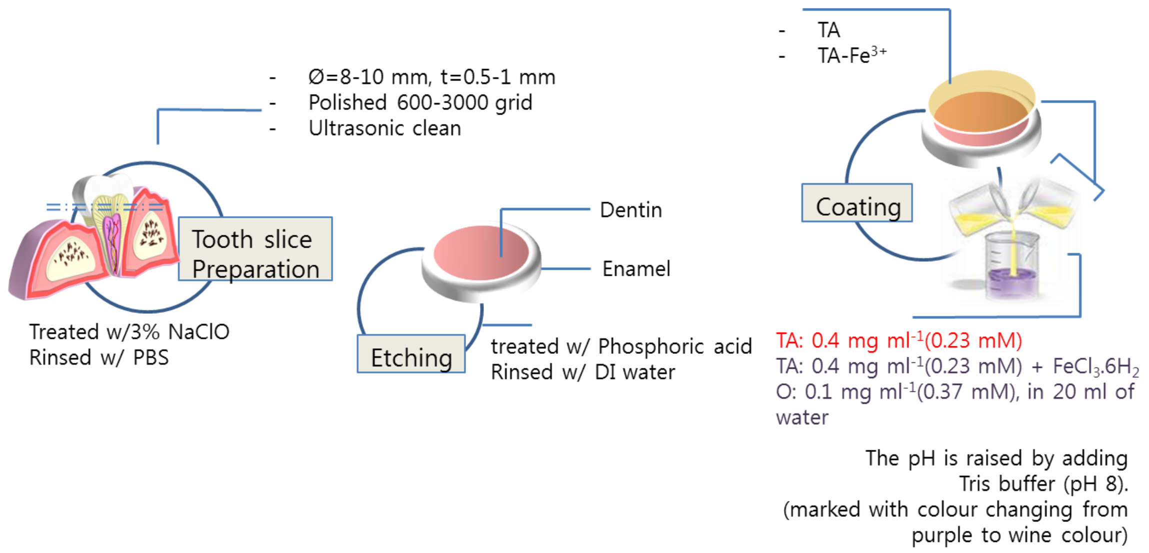

Figure S2. Schematics of tooth slice preparation, tooth slice etching, and tooth slice coating methods.

Gluma treatment[1,2].

The tooth slices were acid-etched with phosphoric acid etching gel for 30 seconds, then rinsed with sufficient DI (deionized) water. Gluma was applied to the tooth slice and then leaved for 60 s. The excess of Gluma was rinsed with DI water.

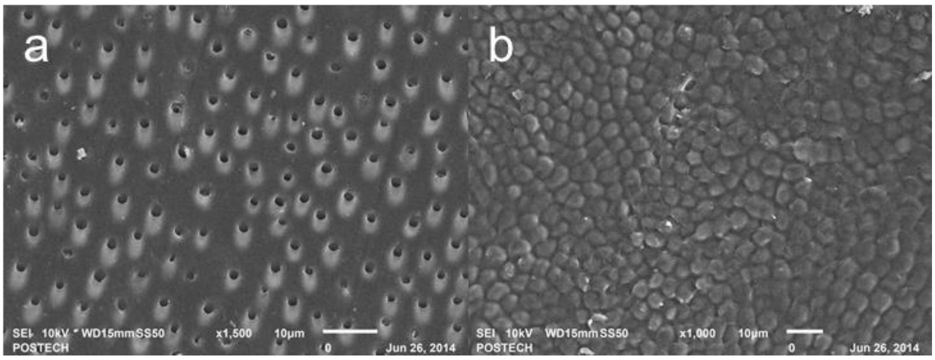

Figure S3. SEM images of (**a**) diluted Gluma-coated and (**b**) original Gluma-coated tooth slices.

XPS analysis on calcium absorption of TA/Fe^{3+}-complex film[3].

X-ray photoelectron spectroscopy (XPS) analysis was conducted with a PHI 5800 ESCA System at 2 × 10^{-6} tor with a monochromatic Al Kα (1486.6 eV) anode (250 W, 10 kV, 27 mA). All binding energies were tuned to the main hydrocarbon peak, C 1s (284.6 eV). All XPS spectra were evaluated using CasaXPS. TA/Fe^{3+}-coated PTFE membrane was immersed in 50 mM Ca_2Cl aqueous solution for 12 h, then fully washed with DI water and acetone. To investigate calcium absorption of TA/Fe^{3+} complex, wide-scan XPS spectra and calcium 2p XPS narrow-scan spectra of the TA/Fe^{3+}-coated PTFE membrane were obtained.

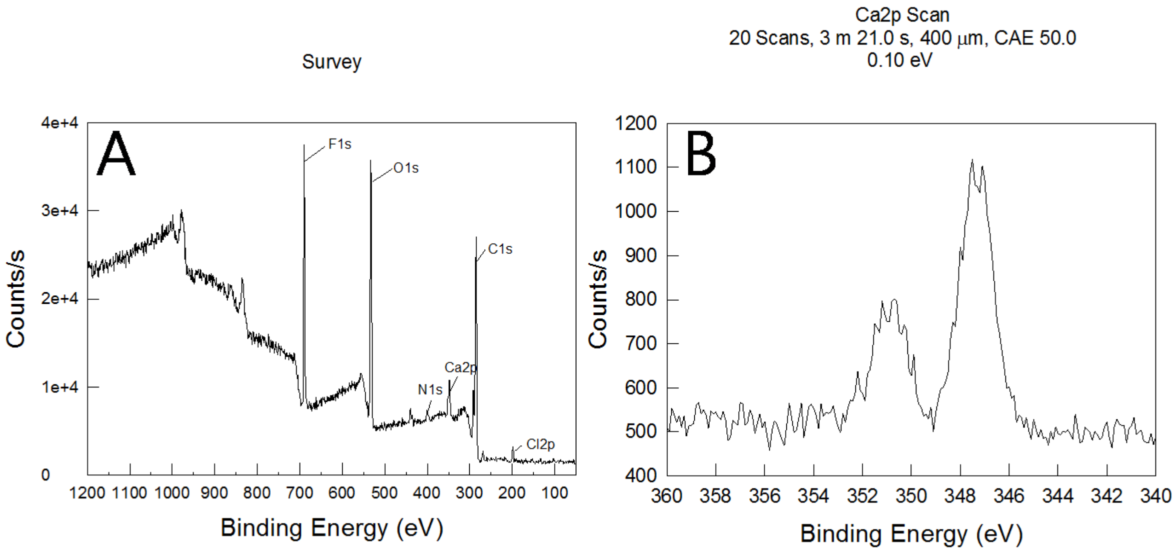

Figure S4. (**a**) Wide scan and (**b**) Ca 2p narrow scan XPS spectra of TA/Fe^{3+} coated polytetrafluoroethylene (PTFE) filter membrane after 12 h immersion in 50 mM $CaCl_2$ buffer.

In the Ca 2p narrow scan XPS spectra of the TA/Fe^{3+} coated polytetrafluoroethylene (PTFE) filter membrane, the notable Ca 2p peaks suggests that TA/Fe^{3+} complex film absorbed calcium ions on the surface.

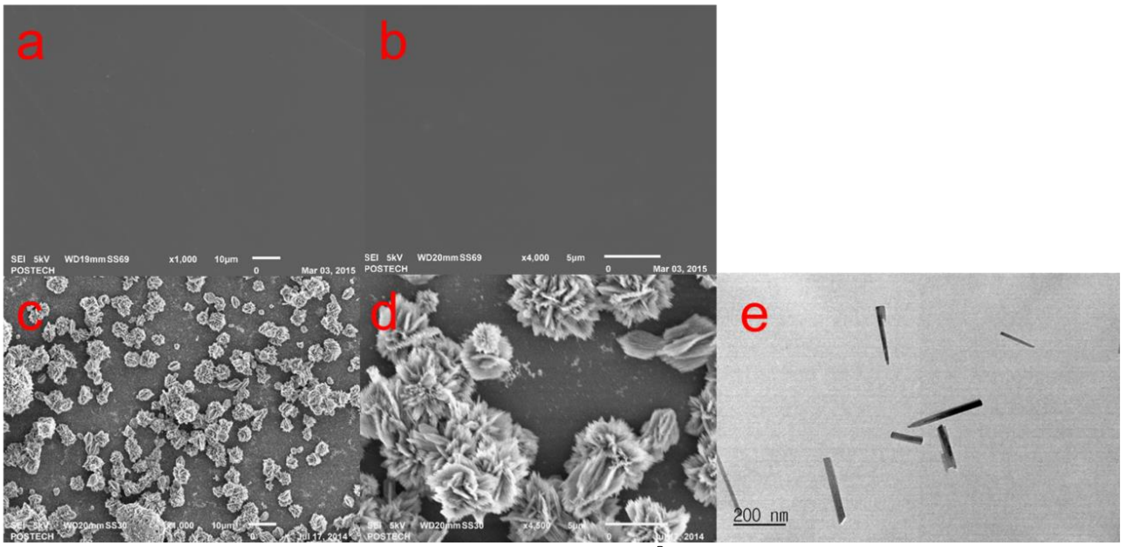

Figure S5. SEM images of (a,b) neat PS and (c,d) TA/Fe^{3+} coated PS films after immersed in artificial saliva; HA minerals grew on TA/Fe^{3+} coated PS. (e) TEM image of fragmented HA minerals that grew on TA/Fe^{3+} coated PS film.

Dentinal fluid flow measurement[4,5].

Dentinal fluid flow measurement consists of three main parts: (1) a glass capillary and photo-sensor to detect the fluid movement, (2) a stepping motor, lead screw, and ball nut to track the fluid movement; and 3) a rotary encoder to record the fluid movement (Supplementary Fig. 6). The water reservoir and the tooth slice was connected at both ends of the water filled glass capillary with an internal diameter of 0.5 mm. A photo transistor detected the movement of an air bubble trapped within the capillary during dentinal fluid flow measurement. The air bubble position that represent fluid flow was tracked by stepping motor, and the rotary encoder translated the air bubble movement to the electric signal. The electric signal was translated into the amount of fluid flow within the dentinal tubules. It was reported that the minimum measurable volume of water movement using this system is approximately 0.196 nL.

A tooth slice sample was connected to a glass capillary by silicone tubing. A hydrostatic pressure of 200 mmH_2O was applied throughout all of the procedures with a water reservoir. The temperature and relative humidity of the environment were ~20 ℃ and ~30%, respectively. After the tooth slice was connected, this instrument underwent a stabilizing time of 10 minutes. Then, the amount of water infiltration in a new tooth slice was first measured for 10 min, and the standard graph of the tooth slice was constructed; the amount of water infiltration vs time. After coating treatment, the amount of water infiltration in the coated tooth slice was again measured for 10 min, and the amount was compared to the standard value.

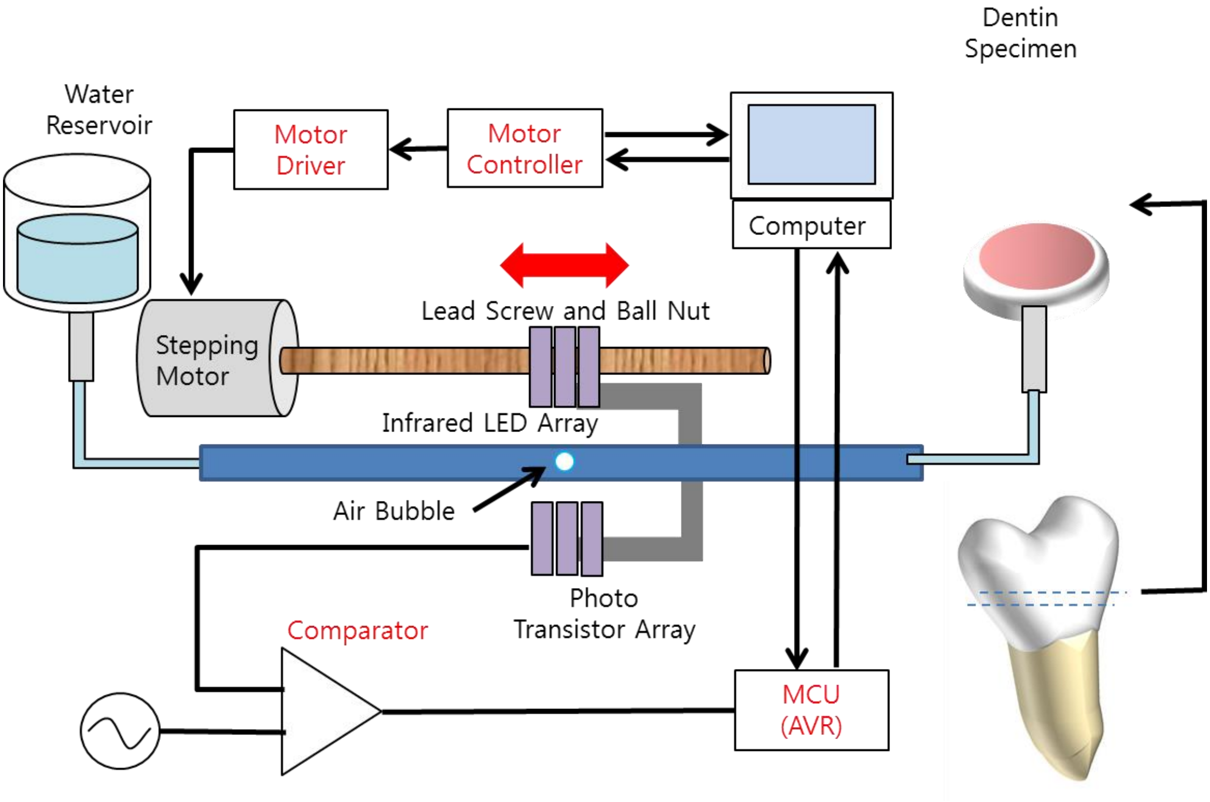

Figure S6. Schematic diagram of dentinal fluid flow (DFF) test.

References

1. Femiano, F. *et al.* Efficacy of diode laser in association to sodium fluoride vs Gluma desensitizer on treatment of cervical dentin hypersensitivity. A double blind controlled trial. *American journal of dentistry* **26**, 214-218 (2013).
2. Schupbach, P., Lutz, F. & Finger, W. Closing of dentinal tubules by Gluma desensitizer. *European journal of oral sciences* **105**, 414-421 (1997).
3. Ryu, J., Ku, S.H., Lee, H. & Park, C.B. Mussel-Inspired Polydopamine Coating as a Universal Route to Hydroxyapatite Crystallization. *Advanced Functional Materials* **20**, 2132-2139 (2010).
4. Kim, S., Kim, E., Kim, D. & Lee, I. The Evaluation of Dentinal Tubule Occlusion by Desensitizing Agents: A Real-time Measurement of Dentinal Fluid Flow Rate and Scanning Electron Microscopy. *Operative dentistry* **38**, 419-428 (2013).
5. Kim, S.-Y., Ferracane, J., Kim, H.-Y. & Lee, I.-B. Real-time measurement of dentinal fluid flow during amalgam and composite restoration. *Journal of dentistry* **38**, 343-351 (2010).
